# Orthopedics: A Systematic Treatise upon the Prevention and Correction of Deformities

**Published:** 1867-04

**Authors:** 


Orthopedics: A Systematic Treatise upon the Prevention and Correction of Deformities. By David Prince, M.D. Philadelphia: Lindsay & Blakiston. 1866.

In the preceding issue of the Examiner, we mentioned the fact that no copy of this work had been sent to us, but inserted in the same number a notice of its merits, furnished by a friend. Since that time, we have received a copy from the publishers, and find it an elegant volume of 240 pages, well illustrated with cuts. It is a good, practical, and reliable work, exactly such as every practitioner needs. For sale by W. B. KEEN & Co., 148 Lake St. Price $3.00.

